# Discovery and functional characterization of a novel PilV variant in a *mcr-1*-positive IncI2 plasmid

**DOI:** 10.1128/spectrum.01825-26

**Published:** 2026-09-30

**Authors:** Chen An, Jie Yang, Silin Liu, Dongyan Shao, Hongwei Zhou, Yuchen Wu, Yang Wang, Rong Zhang, Jianzhong Shen, Congming Wu, Yingbo Shen

**Affiliations:** 1 State Key Laboratory of Veterinary Public Health and Safety, College of Veterinary Medicine, China Agricultural University 34752, Beijing, China; 2 College of Veterinary Medicine, China Agricultural University 34752, Beijing, China; 3 Sanya Institute, China Agricultural University 34752, Sanya, China; 4 The Second Affiliated Hospital Zhejiang University School of Medicine 89681, Hangzhou, China; University of Nebraska-Lincoln, Lincoln, Nebraska, USA

**Keywords:** *mcr-1*, IncI2 plasmid, type IV pilus, shufflon, conjugation

## Abstract

IncI2-type plasmids are major vehicles for the dissemination of *mcr-1*. These plasmids encode a type IV secretion system (T4SS) that mediates conjugative transfer and a type IV pilus (T4P) that facilitates mating-pair formation. Shufflon-mediated diversity of tip adhesin PilV of T4P may contribute to the extensive dissemination of IncI2 plasmids. Herein, we identified a novel shufflon segment forming an active new PilV variant from an IncI2 plasmid, which was named PilV-E and was detected in 127 of 501 (25.35%) complete IncI2 plasmids in public databases. We further demonstrated that strains harboring the single PilV-E variant displayed a distinct functional profile, characterized by both robust biofilm formation and high *Caco-2* adhesion. Compared with the Δ*pilV*-shufflon-*rci* strain, the PilV-E-carrying strain showed increased biofilm formation (OD_590_ from 1.50 to 2.60), increased *Caco-2* adhesion (0.04% to 5.46%), and increased surface motility (1.20 to 1.87 cm/day). Among all variants, PilV-A' showed the strongest cell adhesion (6.00%), whereas PilV-B' showed the greatest motility (2.54 cm/day). Notably, PilV-E significantly restored conjugation frequency compared with the Δ*pilV*-shufflon-*rci* strain and displayed the highest and lowest frequencies when recipients were *Escherichia coli* (5.23 × 10^−3^) and *Acinetobacter baumannii* (4.46 × 10^−8^), respectively. All findings confirm that PilV-E serves as a novel and key tip adhesin of T4P and plays a crucial role in plasmid conjugation, indicating that the diversity of shufflon-mediated PilV variants has been underestimated and providing insight into the molecular basis for the extensive spread of IncI2 plasmids.

**IMPORTANCE:**

The plasmid-mediated colistin resistance gene *mcr-1* has become a major public health concern because it compromises one of the last-line treatments for multidrug-resistant Gram-negative bacterial infections. IncI2 plasmids are among the most important vehicles for the dissemination of *mcr-1*, but the molecular factors that facilitate their efficient transfer remain insufficiently understood. In this study, we identified PilV-E, a novel shufflon-associated type IV pilus tip adhesin encoded by a human-derived IncI2 plasmid, and demonstrated its contribution to bacterial fitness-related phenotypes and conjugative transfer. The prevalence of PilV-E among complete IncI2 plasmids further indicates that this variant has been overlooked in previous studies. These findings expand our understanding of shufflon-mediated PilV diversity and provide new insight into how IncI2 plasmids participate in the efficient dissemination of *mcr-1* among diverse bacterial species, including clinically important pathogens.

## INTRODUCTION

Bacterial antimicrobial resistance has emerged as a major global public health challenge. Colistin, as the last line of defense for treating infections caused by multidrug-resistant Gram-negative bacteria, has garnered significant attention regarding its resistance issues. The plasmid-mediated transferable colistin resistance gene *mcr-1* was first discovered in 2015 (1). Among globally reported *mcr-1*-positive plasmids, over 90.00% belong to the three major incompatibility groups: IncX4, IncI2, and IncHI2. These plasmids primarily facilitate the rapid horizontal dissemination of *mcr-1* through conjugation (2). Among these plasmids, IncI2 was the first plasmid type reported to carry *mcr-1* and is considered the optimal vector for *mcr-1* dissemination. IncI2 plasmids are widely distributed across several sectors (animals, humans, food, and environment) and various Enterobacteriaceae species (3–5). Although China has banned colistin as a feed additive and the prevalence of colistin-resistant *E. coli* has largely decreased thereafter, the proportion of *mcr-1*-positive IncI2 plasmids has increased unexpectedly (from 25.40% to 34.50%) (6, 7). Those data indicate that IncI2 plasmids may be well adapted to diverse bacterial backgrounds and exhibit broad-spectrum transmissibility. *In vitro* competition assays have shown that *E. coli* strains carrying IncI2 plasmids exhibit growth advantages over strains harboring IncHI2 and IncX4 plasmids (8). This may be attributed to the plasmid copy number regulatory factor (PcnR) encoded by IncI2, which binds to *repR* mRNA and downregulates the expression of the replication initiation protein RepA, thereby reducing plasmid copy number to mitigate the fitness cost to the bacterial cell and enhance stable maintenance (9). This mechanism partially explains the ongoing increase in the proportion of IncI2 plasmids carrying *mcr-1* in recent years, underscoring their substantial adaptive advantages.

Plasmid persistence in microbial communities depends not only on low fitness costs and stable maintenance in bacterial hosts but also on efficient conjugative transfer (10, 11). Plasmid conjugation is mediated by the type IV secretion system (T4SS), which enables the direct transfer of plasmids between bacteria (12–14). A recent study reported that IncI2, IncX4, and IncHI2 plasmids all encode a T4SS and exhibit different recipient ranges and conjugative transfer frequencies (5). Furthermore, comprehensive reviews have described the promoting and inhibitory effects of external compounds (such as antibiotics, disinfectants, and metal ions) on plasmid conjugation (15). Beyond external factors, internal factors such as host restriction-modification (RM) systems, CRISPR-Cas systems, and type IV pilus (T4P) could also modulate the conjugation process (16, 17). Studies have demonstrated that the pCT plasmid (similar to R64) lacking the *pilV* gene fails to form complete T4P, resulting in ineffective mating pair formation and ultimately reducing conjugation transfer frequency in liquid media (18). Notably, IncI2 plasmids carry genes encoding a T4P, whereas IncX4 and IncHI2 plasmids lack this system. The presence of T4P may, therefore, facilitate the conjugative transfer of IncI2 plasmids compared with the other two plasmid types.

T4P is encoded by the *pil* gene cluster, in which the *pilV* gene encodes the pilus tip adhesin (19). The C-terminal region of PilV is expressed as a fusion protein with glutathione S-transferase (GST), which binds to lipopolysaccharide (LPS) on the recipient bacterial surface, determining recipient recognition during liquid conjugation transfer (20–23). Notably, the C-terminal structure of PilV is regulated by sequence rearrangement of a region via Rci recombinase, which is referred to as the shufflon system. The shufflon was first discovered in 1986 by Teruya Komano et al. in the IncI1 plasmid R64, consisting of four invertible segments (seven variants), and was located between the site-specific recombinase gene *rci* and the T4P adhesin gene *pilV* (24). This system generates diverse C-terminal segments of the *pilV* gene through rearrangement, thereby producing multiple pilus tip adhesins. These adhesins recognize core oligosaccharide components of LPS on different recipient bacteria, influencing the formation of donor-recipient mating pairs, and thereby regulating plasmid conjugation transfer frequency to expand plasmid transmissibility (25–28). Sekizuka et al. utilized second- and third-generation sequencing technologies to reveal that individual colonies harboring identical IncI2 plasmids contain multiple shufflon segment proportions (A, BD, and C), highlighting remarkable structural diversity (29). It is noteworthy that the shufflon region of IncI2 is not highly conserved, with each plasmid exhibiting a unique shufflon structure. Even between two strains of the same ST type (ST457), MRY15-117 and MRY15-131 plasmids exhibit insertions or deletions of segment C in their shufflons (29). Allard et al. showed that the TP114 plasmid harbors a novel four-segment shufflon architecture, producing eight distinct PilV variants (including seven previously known ones plus a new D variant) (30). These findings indicate that the number of known shufflon segment variants has increased from seven (R64) to eight (TP114), and the actual diversity is likely much greater, with additional unidentified variants still awaiting discovery.

Although prior studies have found eight shufflon segments and sporadically elucidated their potential roles in regulating pilus protein expression and influencing plasmid conjugation transfer, the existence of novel segments and a comprehensive comparison of their biological functions remain to be investigated. To address this issue, we retrieved 115 complete IncI2 plasmid sequences from our laboratory database and identified a novel shufflon segment in an *mcr-1*-positive IncI2 plasmid. Based on that, we comprehensively evaluated the effects on plasmid conjugation transfer and other biological functions for all nine shufflon segments, which provides scientific evidence to better understand the diversity of the shufflon and the molecular basis of transferability of the *mcr-1*-positive IncI2 plasmid.

## RESULTS

### Identification of a novel shufflon segment

**Fig 1:**
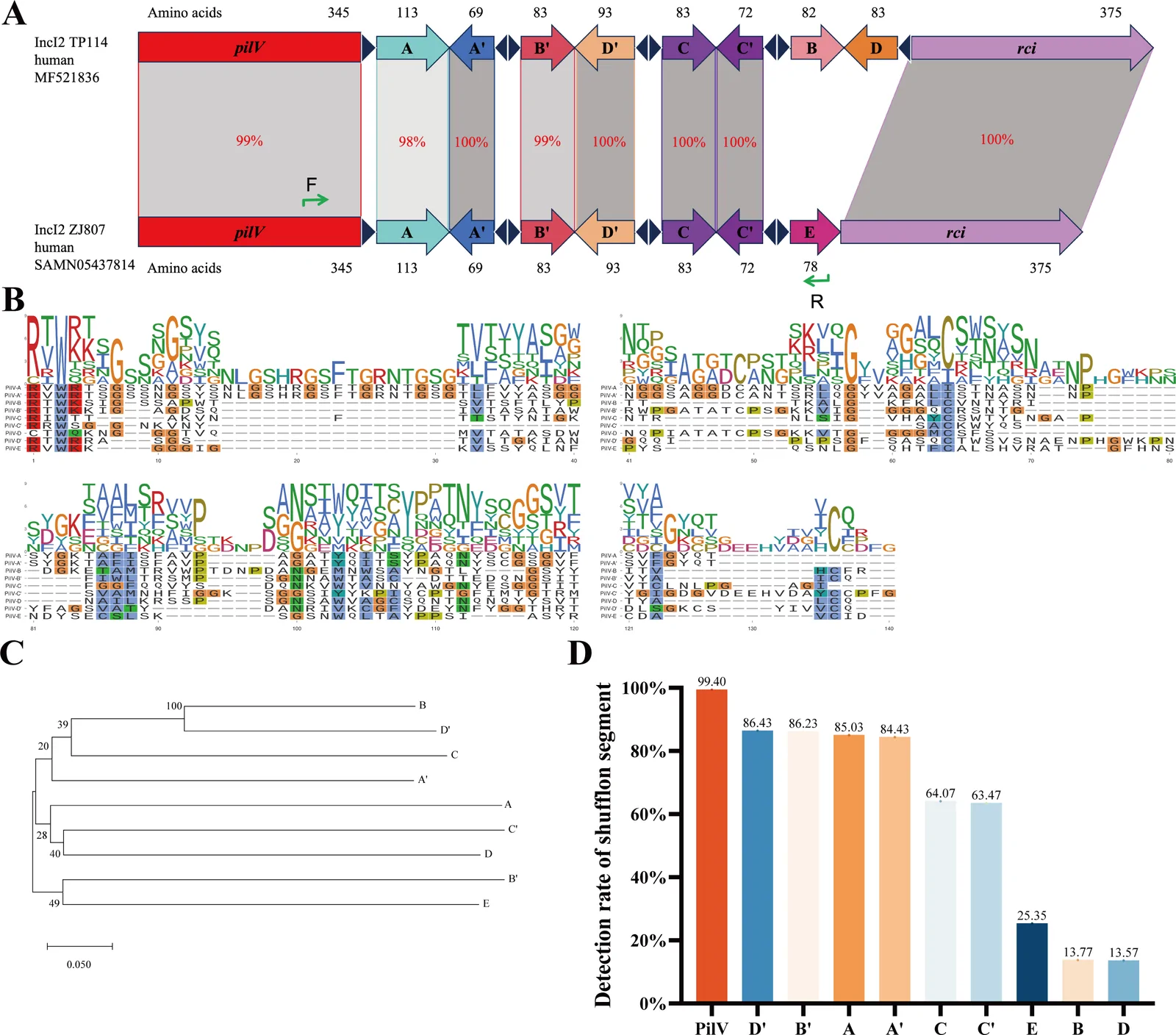
Genetic context, homology, and prevalence of the novel PilV variant. (**A**) The shufflon regions of pTP114 (MF521836) and pZJ807 (SAMN05437814). The colored arrows indicate the transcriptional orientation of each ORF. Shading between the aligned sequences indicates homologous regions, with exact sequence identities shown as red percentages. The amino acid lengths of the protein regions are indicated above and below each compared ORF. PCR primers used for amplifying the shufflon region are shown as green arrows above and below the sequences. (**B**) Multiple sequence alignment of PilV variants derived from plasmid ZJ807. The sequence logos above the alignment indicate residue conservation, with letter height reflecting the relative conservation at each position. Amino acid residues are colored according to their physicochemical properties: hydrophobic residues (A, V, I, L, M, F, W, Y) are shown in blue, polar uncharged residues (S, T, N, Q, C, G) are shown in green, positively charged residues (K, R, H) are shown in red, negatively charged residues (**D and E**) are shown in orange, and proline (P) is shown in purple. Alignment gaps are indicated by dashes. (**C**) Phylogenetic tree of PilV variants derived from plasmid ZJ807. The tree was constructed using the neighbor-joining method in MEGA X software. Bootstrap analysis was performed with 1,000 replicates, and the values were shown at nodes. (**D**) Prevalence of shufflon segment. All sequences were detected using nucleotide, and PilV indicates sequence excluded shufflon segment.

A detailed sequence analysis was performed on the completely sequenced IncI2 plasmids stored in the laboratory (*n* = 115), and one strain was found to carry a novel DNA segment showing non-homology with all eight shufflon segments (Fig. 1A and B). Furthermore, phylogenetic analysis of PilV protein indicated that the novel variant was most closely related to PilV-B' (Fig. 1C). In-depth sequence analysis further revealed that this new segment and the upstream 5′ region of the *pilV* gene together form a complete open reading frame (ORF). This ORF was, therefore, designated as *pilV-*E. Additional analysis showed that *pilV-*E contains the conserved *sfx* sequence (GTGCCAATCCGGTNNTGG), suggesting that it could be regulated by the downstream Rci recombinase and, thus, meets the requirements for sequence recombination within the shufflon region. To investigate this, PCR was conducted and the results showed that the shufflon region is regulated by the Rci recombinase in the wild-type strain with multiple bands in agarose gel electrophoresis (Fig. S1A). Furthermore, by constructing an expression vector for the PilV-E variant, western blot analysis confirmed that this variant can be expressed as a full-length protein (Fig. S1B). To determine the prevalence of the PilV-E variant, the 3′ DNA fragment of *pilV-*E was compared against all complete IncI2 plasmid sequences (*n* = 501) available in the NCBI database. The results showed that 99.40% (498/501) of IncI2 plasmids contain the *pilV* gene and shufflon segments, with *pilV-*E (25.35%, 127/501) ranking seventh in prevalence (Fig. 1D). All these findings indicate that a novel shufflon segment and PilV variant were identified and widely distributed among IncI2 plasmids.

### PilV-E variant exhibits enhanced growth and biofilm formation

**Fig 2:**
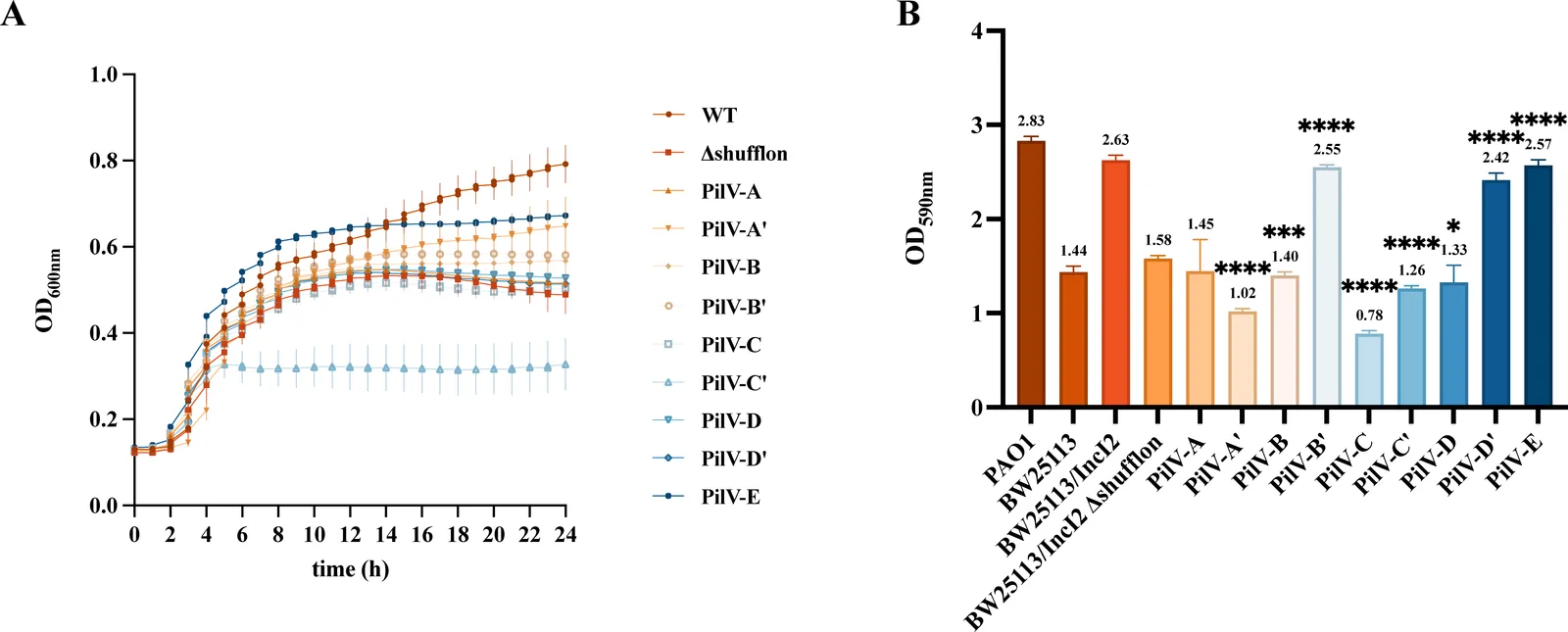
Growth curve and biofilm formation of strains carrying individual PilV variants. (**A**) Growth curves. Bacterial growth was determined by measuring OD_600_ every 1 h for 24 h. Data represent the mean ± SD of three biological replicates (*n* = 3). (**B**) Biofilm formation. Data represent the mean values of three biological replicates, with error bars indicating standard deviation. Statistical analysis was performed using unpaired *t*-tests, with each PilV variant compared with the Δ*pilV*-shufflon-*rci* strain. *P < 0.05, ***P < 0.001, and ****P < 0.0001.

We constructed nine IncI2 plasmid-bearing BW25113 strains, each carrying an individual PilV variant. To determine whether subsequent phenotypic differences were associated with variation in *pilV* expression, we quantified the relative transcription levels of *pilV* using RT-qPCR. No significant differences were detected among the variant-carrying strains when each variant was compared with PilV-A (Fig. S2), suggesting that the observed phenotypic differences were unlikely to be primarily caused by differences in *pilV* transcription. Nevertheless, differences in the stability of PilV variants may still affect their protein levels despite comparable transcription levels. Among those strains, the strain carrying PilV-E exhibited the best growth up to 14 h. After 14 h, the wild-type strain showed a growth advantage, while the PilV-C' strain displayed the poorest growth (Fig. 2A). These results indicate that strains carrying different single PilV variants exhibit distinct growth states.

Promotion of biofilm formation is one of the key functions of T4P. We assessed the impact of different PilV variants on biofilm formation using *Pseudomonas aeruginosa* PAO1 as a positive control. The results showed that PAO1 had the strongest biofilm formation, and introduction of the wild-type IncI2 plasmid significantly enhanced biofilm formation in BW25113. Notably, deletion of the *pilV*-shufflon-*rci* region of IncI2 plasmid markedly reduced the biofilm formation, indicating that the T4P promotes biofilm development with the shufflon region being essential for this process. Among strains carrying individual PilV variants, PilV-E, PilV-B', and PilV-D' significantly increased the ability to form the biofilm and reached levels similar to those of the strain carrying the wild-type IncI2 plasmid and PAO1, whereas other variants had little effect on biofilm formation (Fig. 2B). These results indicate that T4P is critical for promoting biofilm formation in the bacterial strain and that different PilV variants forming different adhesins vary significantly in their biofilm-forming ability.

### PilV variants enhance adhesion of plasmid-bearing bacteria to intestinal epithelial cells

**Fig 3:**
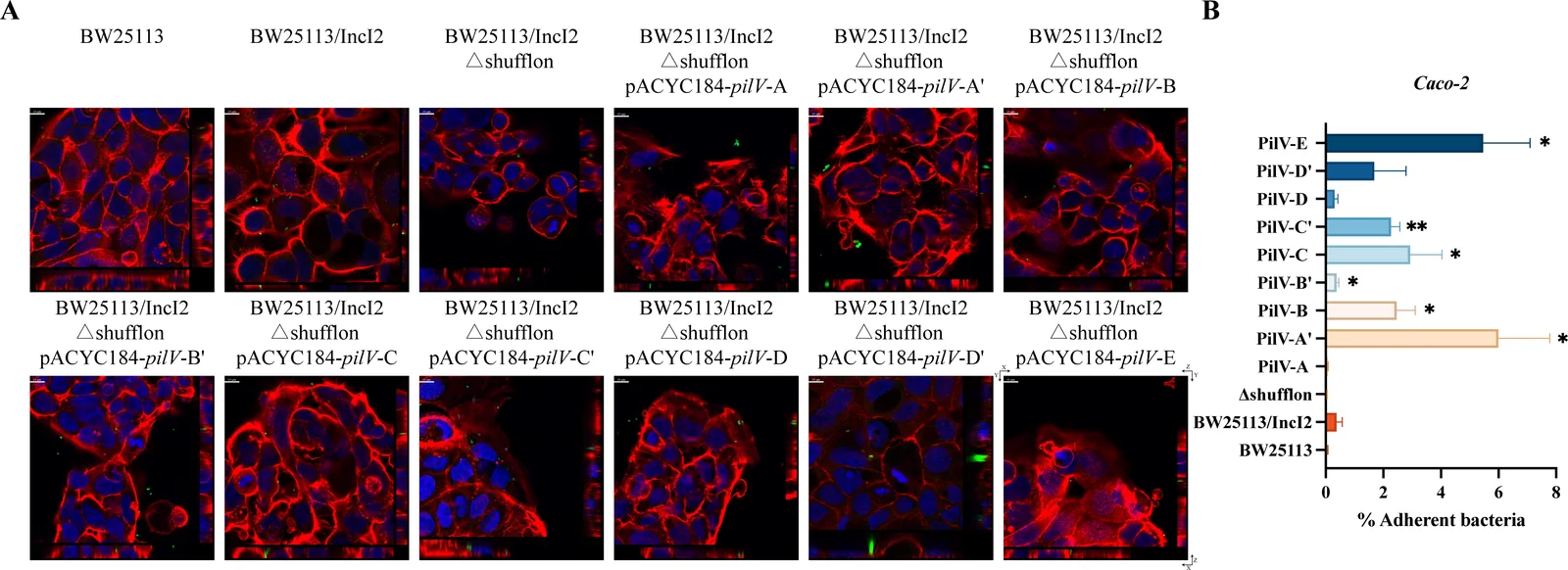
Adhesion of strains carrying different PilV variants to *Caco-2* cells. (**A**) Confocal images showing cell adhesion. Representative confocal images of *Caco-2* cells after incubation with the indicated strains at an MOI of 50 for 3 h. Nuclei were stained with DAPI (blue), F-actin was stained with rhodamine-conjugated phalloidin (red), and bacteria were labeled with a FITC-conjugated *E. coli* serotype O/K polyclonal antibody (green). Scale bars, 10 μm. (**B**) Adhesion rate. Data represent the mean values of three biological replicates, with error bars indicating standard deviation. Statistical analysis was performed using unpaired *t*-tests, with each PilV variant compared with the Δ*pilV*-shufflon-*rci* strain. *P < 0.05 and **P < 0.01.

Cellular adhesion is another important function of T4P. Therefore, we used human colon adenocarcinoma cells *Caco-2* to evaluate the effects of different PilV variants on cellular adhesion. The BW25113 strain exhibited a very low adhesion rate at 0.04%, and the adhesion rate was significantly increased to 0.38% after introduction of the wild-type IncI2 plasmid into BW25113, whereas it was markedly reduced to 0.04% by deleting the *pilV*-shufflon-*rci* region (Fig. 3A and B). After complementation with individual PilV variants, all PilV-carrying strains showed varying degrees of enhanced adhesion to *Caco-2* cells, in which PilV-A' exhibited the strongest adhesion (6.00%), followed by PilV-E (5.46%), PilV-C (2.93%), and PilV-B (2.46%) (Fig. 3B). All findings indicate that T4P enhances bacterial adhesion to *Caco-2* cells, and PilV plays an important role in cellular adhesion with diverse effectiveness.

### PilV variants alter bacterial surface motility

**Fig 4:**
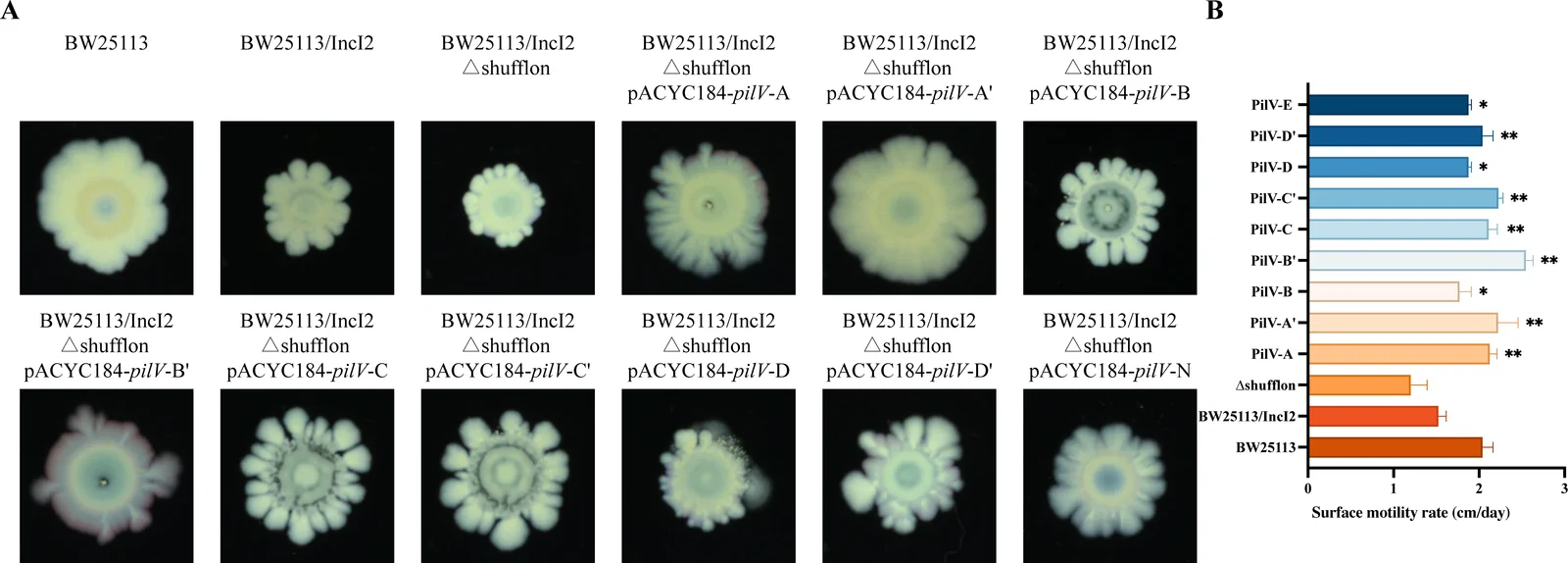
Surface motility of strains carrying different PilV variants. (**A**) Bacterial motility images. (**B**) Surface motility rates. Data represent the mean values of three biological replicates, with error bars indicating standard deviation. Statistical analysis was performed using unpaired *t*-tests, with each PilV variant compared with the Δ*pilV*-shufflon-*rci* strain. *P < 0.05 and **P < 0.01.

T4P can mediate surface motility in plasmid-bearing bacterial strains; thus, we conducted an assessment of bacterial surface motility. Compared with the *pilV*-shufflon-*rci* deletion strain (1.20 cm/day), all strains carrying individual PilV variants exhibited increased motility, in which PilV-B' showed the highest motility (increased from 1.20 to 2.54 cm/day), and PilV-E also showed a significant increase in the motility-zone diameter (increased from 1.20 to 1.87 cm/day) (Fig. 4B). These findings suggest that PilV variants alter the pilus-mediated surface motility and show greater motility than the Δ*pilV*-shufflon-*rci* control.

### PilV plays an important role in IncI2 plasmid conjugation

**Fig 5:**
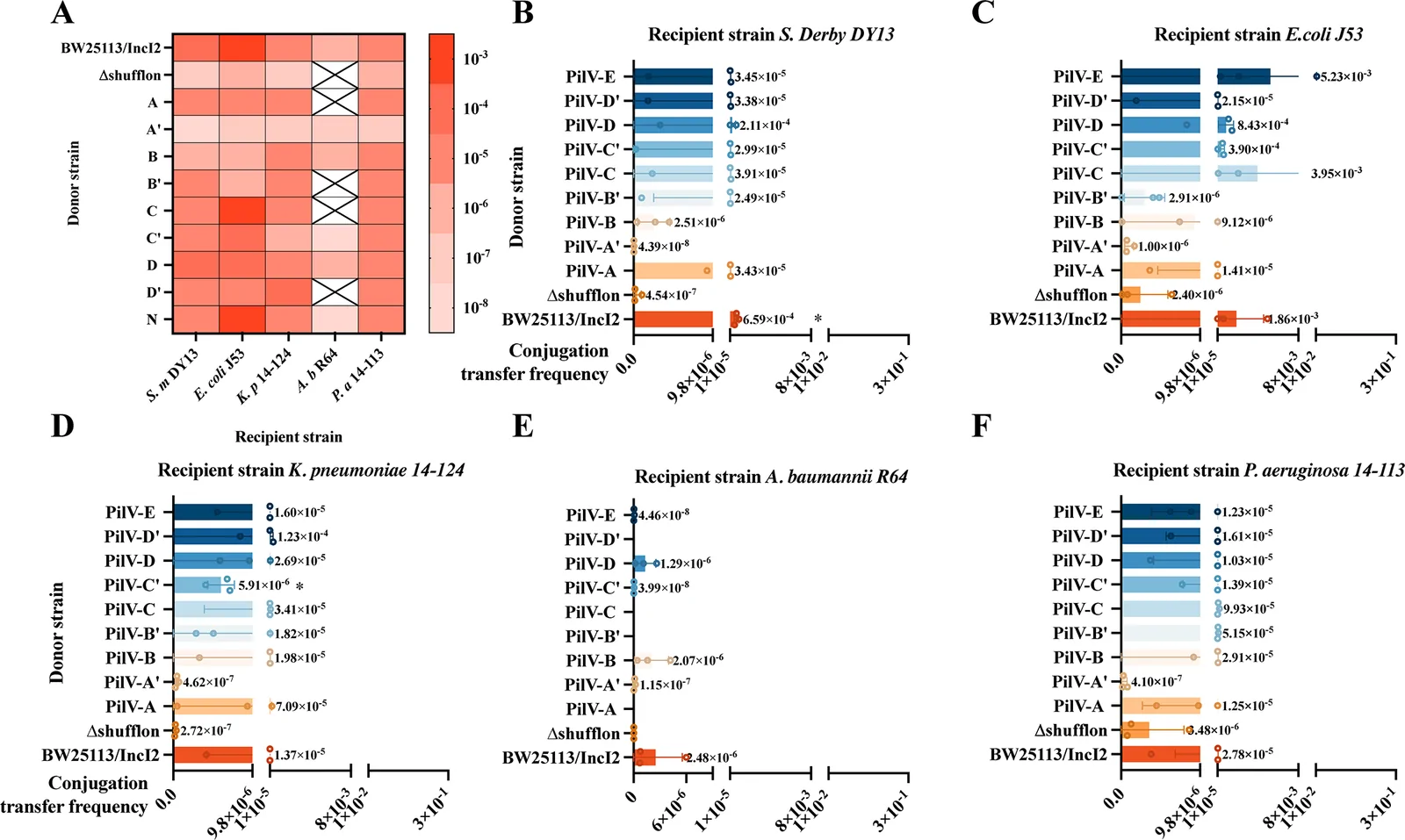
Conjugative frequency of BW25113 carrying different PilV variants. (**A**) Heatmap of the conjugative frequency of BW25113 carrying the wild-type IncI2 plasmid, Δ*pilV*-shufflon-*rci* mutant, and nine individual PilV variants to different recipient strains. Cross marks indicate that no transconjugants were detected. (**B**) *S*. Derby. (**C**) *E. coli*. (**D**) *K. pneumoniae*. (**E**) *A. baumannii*. (**F**) *P. aeruginosa*. Data were the mean values of three replicates (*n* = 3), with error bars indicating standard deviation. Statistical analysis was performed using unpaired *t*-tests, with each PilV variant compared with the Δ*pilV*-shufflon-*rci* strain. *P < 0.05.

We compared the conjugative abilities and frequency of donor strains carrying individual PilV variants with five clinically common and high-risk recipient bacteria including *Escherichia coli*, *Klebsiella pneumoniae*, *Salmonella enterica* serovar Derby, *Pseudomonas aeruginosa*, and *Acinetobacter baumannii*. The wild-type BW25113/IncI2 strain transferred the plasmid to all five recipient strains, with conjugative frequency ranging from 1.00 × 10**^−^**^5^ to 1.00 × 10**^−^**^3^ (Fig. 5A). The conjugative frequency decreased markedly after deletion of the *pilV*-shufflon-*rci*, ranging from 1.00 × 10**^−^**^8^ to 1.00 × 10**^−^**^6^; in particular, no transconjugants were detected when the recipient was *Acinetobacter baumannii*. Donor strains with individual PilV variants displayed recipient-dependent differences in conjugation ability (Fig. 5B through F). Among all PilV variants, the strain carrying the PilV-A' variant exhibited the lowest conjugative frequency for each of the five recipient species. For the PilV-B-carrying strain, conjugation frequency varied depending on the recipient species, with the highest conjugative frequency in *Acinetobacter baumannii* but the lowest in *Salmonella* (Fig. 5B through F). In terms of the PilV-E variant, the strain carrying it had the highest transfer frequency to *E. coli* (5.23 × 10**^−^**^3^) and the lowest to *A. baumannii* (4.46 × 10^−8^). These findings indicate that PilV plays an important role in IncI2 plasmid conjugation, and the shufflon system may regulate conjugation efficiency by modulating the diversity of PilV variants, thereby influencing donor–recipient interactions.

### IncI2 plasmid confers higher conjugative frequency in *E. coli* recipients

**Fig 6:**
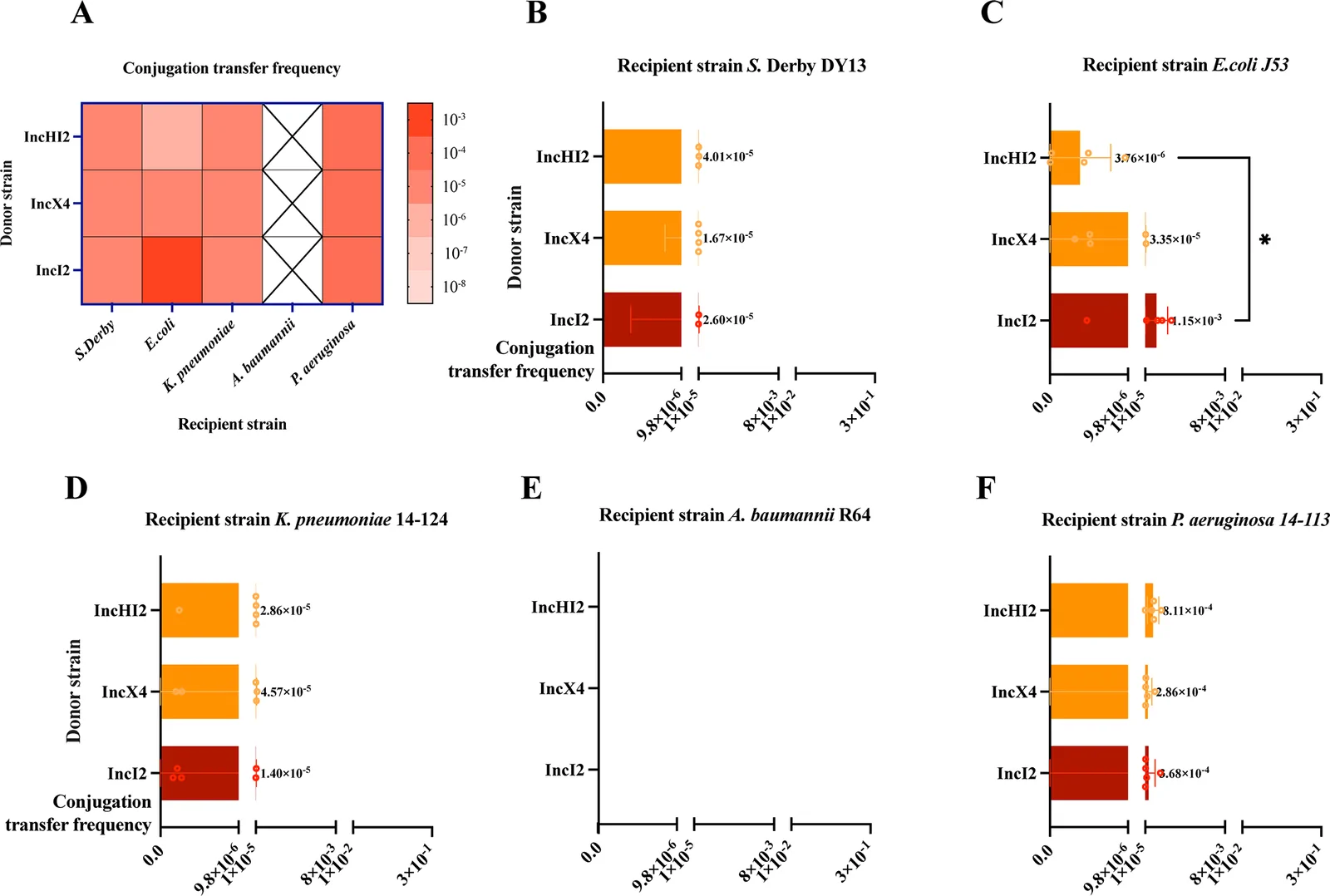
Conjugative frequency of *E. coli* carrying IncI2, IncX4, and IncHI2 plasmids. (**A**) Heatmap of the conjugative frequency of wild-type *E. coli* carrying IncI2, IncX4, and IncHI2 plasmids to different recipient strains. Cross marks indicate that no transconjugants were detected. (**B**) *S*. Derby. (**C**) *E. coli*. (**D**) *K. pneumoniae*. (**E**) *A. baumannii*. (**F**) *P. aeruginosa*. Data represent five independent donor strains for each plasmid type (*n* = 5), with error bars indicating standard deviation. Absence of data points indicates that no transconjugants were detected. Statistical analysis was performed using the Kruskal–Wallis test followed by Dunn’s multiple comparisons test with correction for multiple testing; *adjusted *P* < 0.05.

We further examined whether wild-type IncI2 plasmids exhibit a recipient-dependent transfer advantage over IncX4 and IncHI2 plasmids. *E. coli* strains carrying IncI2 (*n* = 5), IncX4 (*n* = 5), and IncHI2 (*n* = 5) plasmids were used as donors and mated with five recipient strains, including *E. coli*, *S*. Derby, *K. pneumoniae*, *A. baumannii*, and *P. aeruginosa*, respectively. Conjugative frequencies varied markedly among different plasmids and recipient species (Fig. 6A). In *Salmonella* Derby, all three plasmids showed relatively low transfer frequencies, with IncHI2 (2.41 × 10^−5^) slightly higher than IncX4 (1.33 × 10^−5^) and IncI2 (1.04 × 10^−5^) without statistical significance (Fig. 6B). In *E. coli* recipients, the IncI2 plasmid exhibited the highest conjugation frequency (mean 1.15 × 10^−3^), while IncX4 (3.35 × 10^−5^) and IncHI2 (3.76 × 10^−6^) plasmids showed lower conjugation frequencies (Fig. 6C). In *K. pneumoniae*, IncX4 showed the highest average transfer frequency (3.88 × 10^−5^), followed by IncHI2 (2.86 × 10^−5^) and IncI2 (1.44 × 10^−5^) (Fig. 6D). No transconjugants were observed in *A. baumannii* (Fig. 6E). In *P. aeruginosa*, IncHI2 (8.10 × 10^−4^) showed the highest mean value, followed by IncI2 (3.67 × 10^−4^) and IncX4 (2.87 × 10^−4^) (Fig. 6F). Findings suggest that IncI2 displayed a transfer advantage in only *E. coli*.

### Structural comparison of predicted PilV variants

We used AlphaFold 3 to predict the three-dimensional structures of the PilV variants. All variants exhibited highly similar elongated, ladle-shaped architectures. All PilV structures were displayed in the same orientation to facilitate comparison of the conserved N-terminal region and the shufflon-derived variable C-terminal region. The conserved 5′ region of the *pilV* gene encodes the N-terminal α-helical stem and the shared core of the head domain, whereas the shufflon-derived 3′ variable region encodes the distal C-terminal adhesin extension. Structural differences among the PilV variants were primarily confined to this variable C-terminal extension. Enlarged views of the C-terminal variable regions are provided in Fig. S3 for clearer comparison. Combined with the experimental results described above, this suggests that PilV proteins may achieve functional diversity through localized conformational changes while maintaining an overall functional structural framework.

## DISCUSSION

Our study has identified a novel shufflon segment that could be regulated by Rci recombinase and form a novel PilV variant (named PilV-E) with the conserved N-terminal region of PilV. The classic shufflon system, first described in 1986–1987 in the IncI1 plasmid R64, consists of four invertible segments (A, B, C, D) that can generate seven PilV variants (A, A', C, C', B, B', D') (24–26). Subsequent studies revealed structural heterogeneity in the IncI2 shufflon (29), demonstrating high intra-clonal diversity capable of producing novel segment combinations. Stosic et al. reported that IS*Ecp1* insertion caused variant interruption and loss of new segment (31). Allard et al. demonstrated that the TP114 plasmid harbored a novel four-segment shufflon architecture, yielding eight PilV variants—including the seven previously known variants plus a new D variant—thereby expanding the shufflon diversity (30). The discovery of the novel variant in this study represents the latest example, confirming the recombinase-driven evolutionary diversification of shufflon and PilV-mediated pilus, which further gives new insight into the diversity of shufflon and associated PilV adhesin and suggests an underestimated diversity of this region. Global analysis of the IncI2 plasmid database further revealed a detection rate of 25.35% (127/501) for the PilV-E variant, with widespread distribution across multiple bacterial species (e.g., *Escherichia coli*, *Salmonella enterica*) and across different isolation sources (human, animal, and environmental samples) (Table S1 and S2). This relatively high prevalence indicates that PilV-E is not an isolated or recently emerged variant but has already demonstrated significant adaptive fitness in resistance plasmids from diverse global sources.

Given that the PilV adhesin is a critical component for T4P and plays an important role in several phenotypes of plasmid-bearing bacteria, this study systematically investigated the effects of all PilV variants, including the newly identified PilV-E variant, on different bacterial functions such as growth, biofilm formation, cellular adhesion, bacterial motility, and also plasmid transfer (19, 32–35). As a type IV pilus tip adhesin, PilV-E exerts multiple physiological effects—including motility, adhesion, and biofilm formation—that influence bacterial growth and adaptability. These effects suggest that this adhesin variant may facilitate bacterial migration, adhesion to intestinal epithelial cells, surface colonization, and biofilm-associated persistence. The early growth advantage of strains carrying the single PilV-E variant, together with the later competitiveness of wild-type strains, further suggests that shufflon-mediated PilV diversification may provide functional flexibility under different conditions, therefore indirectly increasing opportunities for conjugation transfer and the spread of resistance plasmids.

**Fig 7:**
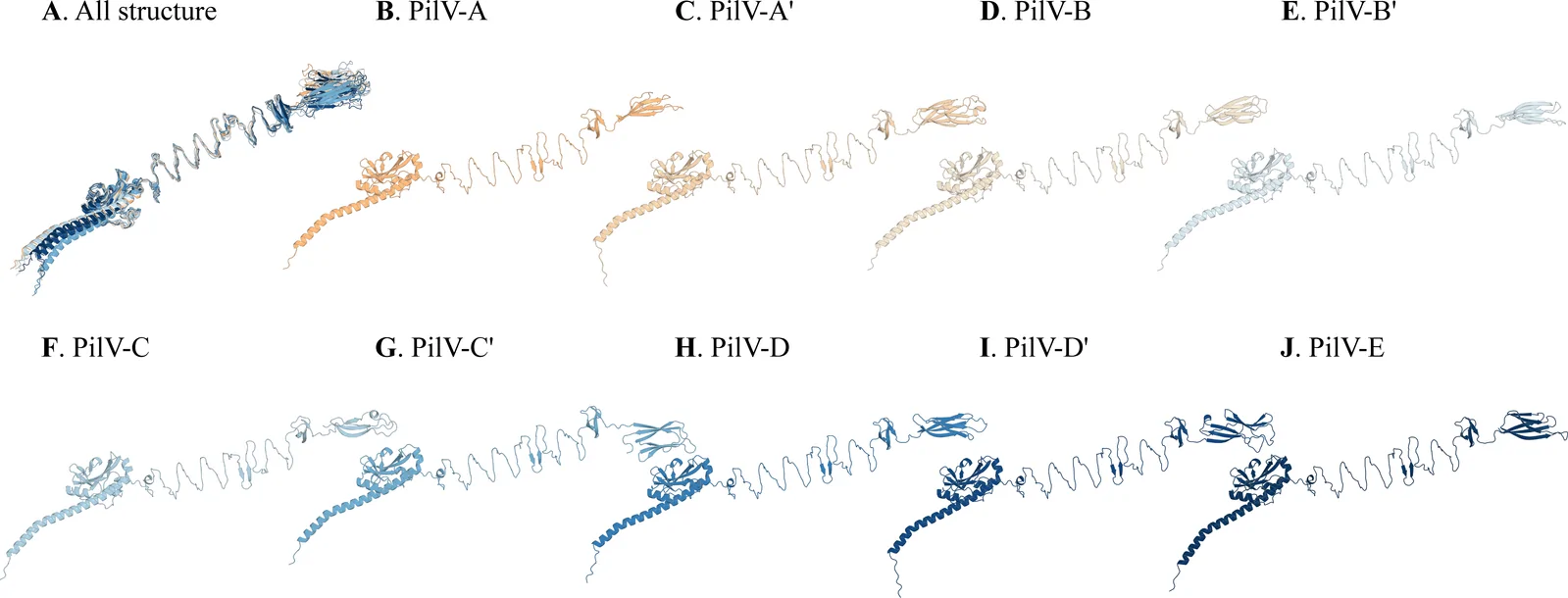
Predicted structures of PilV variants generated using AlphaFold 3. (**A**) Structural superposition of all PilV variants; (**B**) PilV-A; (**C**) PilV-A'; (**D**) PilV-B; (**E**) PilV-B'; (**F**) PilV-C; (**G**) PilV-C'; (**H**) PilV-D; (**I**) PilV-D'; (**J**) PilV-E. All structural models were predicted using AlphaFold 3 and are displayed in the same orientation to facilitate comparison of the conserved and variable regions.

The emergence of novel variants and the dynamic changes in the shufflon region can broaden the recipient range and enhance transmission efficiency. The variable C-terminal region of PilV serves as a key recognition domain that modulates affinity for lipopolysaccharide (LPS) on the recipient bacterial cell surface, thereby enabling recipient-specific recognition and recipient-range expansion. The role of the PilV C-terminal region in recipient recognition is further supported by previously reported C-terminal truncation and substitution experiments showing that replacement of the variable C-terminal region with a FLAG tag abolished adhesion function and nearly eliminated liquid conjugation transfer (30). Structural modeling further revealed that the variable C-terminal region of the PilV-E variant exhibits distinct structural and conformational differences compared with classical variants, thereby altering the interaction sites of PilV with recipient bacterial surface molecules and suggesting a possible structural basis for its enhanced recognition and adhesion capability toward recipient bacteria (Fig. 7). More importantly, in complex microbial communities, shufflon-mediated dynamic switching can confer greater ecological adaptability, allowing strains to rapidly adjust recipient recognition strategies under different bacterial or environmental pressures (29, 30). Conjugation transfer experiments in this study showed that strains carrying the single PilV-E variant exhibited significantly restored conjugation efficiency compared with the *pilV*-shufflon-*rci* deletion strain although the degree of restoration varied among variants, with most frequencies remaining lower than that of the wild-type. Thus, PilV variants not only serve as critical adhesins for donor–recipient recognition and stable contact during liquid conjugation, but their diversity also markedly enhances the dissemination potential of resistance plasmids. Conjugation experiments using clinical strains showed that IncI2 plasmids exhibited the highest conjugation frequency when the recipient was *E. coli*, which may result from the unique T4P encoded by IncI2 plasmids and partially explain the high prevalence of *mcr-1*-carrying IncI2 plasmids in *E. coli* after the ban on colistin as a feed additive.

This study primarily focused on the impact of the single PilV-E variant under fixed expression conditions on IncI2 plasmid dissemination and bacterial fitness-associated phenotypes. However, in natural environments, IncI2 plasmids typically undergo reversible shufflon-mediated rearrangement of PilV, generating multiple tip adhesin configurations. Therefore, this work did not directly evaluate the regulatory effects of shufflon dynamic rearrangement on conjugation efficiency and recipient range under different environmental conditions—an important direction for future exploration. Previous studies have indicated that shufflon structures in *mcr-1*-carrying IncI2 plasmids consist of three segments (A, BD, and C), with different permutations coexisting even at the single-colony level, suggesting that this system may enhance the ecological adaptability of plasmids through shufflon regulation (29). Nevertheless, the contribution of different shufflon configurations to plasmid transmission dynamics in real or simulated ecological settings remains understudied. With the advancement of long-read sequencing technologies and single-colony analysis methods, future research is expected to precisely characterize shufflon rearrangement patterns and their environmental influences without altering the overall genetic background and to further quantify their effects on conjugation behavior under diverse bacterial species and environmental stresses.

In summary, PilV-E is not only a structurally and functionally intact novel adhesin variant but also exhibits unique advantages in early growth competitiveness, adhesion to intestinal epithelial cells, biofilm formation, and plasmid conjugation transfer. It may represent a key determinant in the adaptive dissemination and colonization processes of IncI2 plasmids. Although this study has validated its multifaceted functions through *in vitro* experiments, its influence on plasmid transmission dynamics under external factors and real ecological conditions requires further elucidation. Importantly, the shufflon system provides structural diversity and transmission plasticity to bacteria through dynamic regulation of PilV variants. The discovery of PilV-E further underscores the biological significance of this system in the dissemination and colonization of resistance plasmids.

## MATERIALS AND METHODS

### Bacterial culture

Strains were cultured in Luria-Bertani (LB) broth (Sigma-Aldrich, St. Louis, MO, USA) at 37°C with shaking at 220 rpm or on LB agar plates (Sigma-Aldrich, St. Louis, MO, USA) at 37°C. For conjugation experiments and genetic manipulations, antibiotics were added at the following concentrations: polymyxin B: 2 mg/L; ampicillin: 100 mg/L; meropenem: 2 mg/L; chloramphenicol: 50 mg/L; sodium azide: 100 mg/L. All strains were stored in 20% glycerol at −80°C.

### Bioinformatics analysis

Genomic and plasmid sequences were obtained from the laboratory database and NCBI GenBank database. Nucleotide multiple sequence alignments of shufflon segments were performed using BLASTn (36). Amino acid sequences of PilV variants were aligned using MAFFT version 7 (37), and the resulting alignment was visualized using ggmsa version 1.16.0 (38). A phylogenetic tree of PilV variants derived from pZJ807 (SAMN05437814) was generated using the Neighbor-Joining method in MEGA X software (Molecular Evolutionary Genetics Analysis, USA) (39, 40).

### Construction of plasmids and strains

Bacterial strains, plasmids, and primers used in this study are listed in Table S3 and S4. Mutants of IncI2 plasmids were generated using the λ-Red recombination system. Briefly, the replacement template was amplified from pKD3 using primers *pilV*-del-F/R or RCI-del-F/R and electroporated into competent *E. coli* BW25113 cells carrying the IncI2 plasmid, replacing the *pilV*-shufflon-*rci* region or the *rci* gene with a *cat* cassette, respectively. The *cat* cassette was subsequently excised using pCP20-Spe, and the resulting markerless deletion mutants were confirmed by PCR and Sanger sequencing using primers ∆*pilV*-yan-F/R and ∆RCI-yan-F/R, respectively. The wild-type IncI2 plasmid and its derivative Δ*pilV*-shufflon-*rci* plasmid and Δ*rci* plasmids were then electroporated into BW25113.

Wild-type fragments were PCR-amplified from the 3′ end of *pilV* carrying different shufflon segments from the wild-type IncI2 plasmid using pAC-*pilV*-n-F/R primers. The pACYC184 backbone was amplified using pACYC184-F/R primers. Purified wild-type and mutant PCR products were cloned into the pACYC184 backbone to generate a series of pACYC184-*pilV* complementation plasmids carrying different wild-type or individual *pilV* variants. All mutants and vectors were confirmed by PCR, and the primers were listed in Table S4.

### Antimicrobial susceptibility testing

Minimum inhibitory concentrations (MICs) were determined by the broth microdilution method in cation-adjusted Mueller–Hinton broth (CAMHB; Oxoid, Basingstoke, UK) in accordance with CLSI M07, 12th edition (41). The antimicrobial agents and concentration ranges tested were as follows: colistin, 0.125–32 μg/mL (Sigma-Aldrich, St. Louis, MO, USA); meropenem, 0.016–64 μg/mL (Sigma-Aldrich, St. Louis, MO, USA); and chloramphenicol (Sigma-Aldrich, St. Louis, MO, USA), 0.25–256 μg/mL. Twofold serial dilutions of the antimicrobial agents were prepared in-house in sterile 96-well polystyrene microplates (Corning Incorporated, Corning, NY, USA). The final bacterial inoculum was 5 × 10^5^ CFU/mL, and the plates were incubated at 35 ± 2°C for 18 h. The MIC was defined as the lowest antimicrobial concentration that completely inhibited visible bacterial growth. *E. coli* ATCC 25922 was used as the quality control strain. Quality-control results were evaluated according to CLSI M100, 36th edition (42). Because MIC testing was performed solely to determine antimicrobial concentrations for transconjugant selection, clinical susceptibility categories were not assigned. BW25113 strains carrying the wild-type IncI2 plasmid or the Δ*pilV*-shufflon-*rci* IncI2 plasmid complemented with individual PilV variants, together with *E. coli* J53, *S*. Derby DY13, *K. pneumoniae* 14–124, *P. aeruginosa* 14–113, and *A. baumannii* R64 were subjected to antimicrobial susceptibility testing to determine the appropriate concentrations of corresponding antimicrobial agents used for transconjugant selection.

### Western blot analysis

The plasmid pET28-His-SUMO was amplified using pET28-V-F/R primers. The sequences of *pilV*-E and *pilV*-D' were amplified from IncI2 plasmid using *pilV* protein-n-F/R primers and cloned into pET28-His-SUMO to construct expression vectors pET28-His-SUMO-*pilV*-E and pET28-His-SUMO-*pilV*-D'. Single colonies of *E. coli* BL21(DE3) containing pET28-His-SUMO-*pilV*-E or pET28-His-SUMO-*pilV*-D' were cultured overnight and then inoculated at a 1:100 dilution into 20 mL LB broth with 50 mg/L kanamycin, grown to OD_600_ = 0.60–0.80 at 37°C, 220 rpm. IPTG (Sigma-Aldrich, St. Louis, MO, USA) was added to a final concentration of 0.1 mM for 14–16 h induction at 16°C, 220 rpm. Cells were harvested by centrifugation at 4°C, 2,000 × *g* for 30 min, resuspended in 6 mL lysis buffer, and lysed by sonication. After centrifugation, supernatants were collected, and protein concentrations were determined by BCA Protein Assay Kit (Beyotime Biotechnology, Shanghai, China). Samples (30–50 μg) were separated by SDS-PAGE using 10% polyacrylamide gels, transferred to PVDF membranes (Millipore, Billerica, MA, USA), blocked with 5% skim milk in TBST (EpiZyme, Shanghai, China) for 1 h at room temperature, and incubated with primary antibodies overnight at 4°C: mouse anti-6 × His (Proteintech, Wuhan, China; Cat. No. 66005-1-Ig) and mouse anti-GAPDH (Abcam, Cambridge, UK; Cat. No. ab125247). Membranes were washed, incubated with HRP-conjugated goat anti-mouse IgG (1:5,000, Beyotime A0216) for 1 h at room temperature, and developed using ECL reagents (Thermo Fisher Scientific, Waltham, MA, USA). GAPDH was used as a loading control.

### Growth curve assay

Strains were cultured on LB agar containing 2 mg/L polymyxin B, and a single colony was inoculated into LB broth and cultured overnight at 37°C, 220 rpm. Overnight cultures were diluted 1:100 into fresh LB and grown to logarithmic phase. Cultures were adjusted to OD_600_ = 0.50, diluted 1:100, and 200 μL of the culture was added to 96-well plates with at least three biological replicates per strain. OD_600_ was measured every 1 h for 24 h using an Infinite M200 plate reader (Tecan, Männedorf, Switzerland).

### Biofilm formation assay

Overnight cultures in TSB (Sigma-Aldrich, St. Louis, MO, USA) were adjusted to 0.5 McFarland standard, and 200 μL of the culture was added to 96-well plates and incubated statically at 37°C for 48 h. After incubation, supernatants were removed, and the wells were gently washed 2–3 times with sterile 1× phosphate-buffered saline (Gibco, Thermo Fisher Scientific, Waltham, MA, USA). The sample was fixed with 100–200 μL methanol (Sigma-Aldrich, St. Louis, MO, USA) for 10 min, stained with 1% Crystal Violet Staining Solution (Solarbio, Beijing, China) for 10 min, washed, and air-dried. Crystal violet was solubilized with 100 μL of 30% acetic acid (Sigma-Aldrich, St. Louis, MO, USA), and absorbance was measured at OD_590_. At least three biological replicates per strain were analyzed.

### Surface motility assay

Cultures at logarithmic phase were adjusted to the same OD_600_, and 10 μL of each culture was spotted onto motility agar. The motility agar contained 10 g·L^−1^ tryptone, 5 g·L^−1^ sodium chloride, 8 g·L^−1^ glucose, and 2.5 g·L^−1^ agar, corresponding to an agar concentration of 0.25% (wt/vol). Plates were incubated at 37°C, and diameters of motility zones were measured at 12 and 36 h, respectively. Motility rate was calculated as the increase in colony diameter between 12 h and 36 h divided by the incubation time.

### Cell adhesion assay and confocal imaging

Human colorectal adenocarcinoma *Caco-2* cells were cultured at 37°C in a humidified incubator with 5% CO_2_. The cells were incubated with bacteria at a multiplicity of infection (MOI) of 50 (50 bacteria per cell). After 3 h, non-adherent bacteria were removed by washing three times with PBS. Cells were lysed with 1% Triton X-100 in PBS, and the lysates were serially diluted and plated on selective media for counting. Three biological replicates were performed. In parallel, bacterial adhesion was visualized by confocal fluorescence microscopy. Cell nuclei were stained with DAPI (Beyotime Biotechnology, Shanghai, China), F-actin was stained with rhodamine-conjugated phalloidin (Invitrogen, Thermo Fisher Scientific, USA), and bacteria were labeled with a FITC-conjugated anti-*Escherichia coli* serotype O/K polyclonal antibody (Invitrogen, Thermo Fisher Scientific, USA; Cat. No. PA1-73033). Fluorescence images were acquired using a Leica TCS SP8 laser-scanning confocal microscope (Leica Microsystems, Germany). Fluorescence signals from DAPI, rhodamine, and FITC were acquired sequentially using the corresponding excitation lasers and emission detection channels to minimize spectral crosstalk. Images were acquired at a resolution of 2,048 × 2,048 pixels, and Z-stack images were collected with a z-step size of 0.22 μm. For each fluorescence channel, identical acquisition parameters were maintained across all experimental groups.

### Plasmid conjugation assay

*E. coli* J53, *S*. Derby DY13, *K. pneumoniae* 14–124, *P. aeruginosa* 14–113, and *A. baumannii* R64 were used as recipient strains, and the donor strains were BW25113-IncI2 and its derivatives. Before conjugation, single colonies of donor and recipient strains were separately inoculated into antibiotic-free LB broth and cultured at 37°C with shaking at 220 rpm until the exponential growth phase. The donor and recipient cultures were then adjusted to an OD_600_ of 0.1 with fresh antibiotic-free LB broth and mixed at equal volumes. The mating mixtures were incubated statically at 37°C for 2 h. After mating, the mixtures were serially diluted and spread-plated onto selective LB agar containing appropriate antibiotics to enumerate recipients and transconjugants. Conjugation frequency was calculated as the number of transconjugants divided by the number of recipients. Thirty to 50 colonies were randomly picked for Autof MS1000 MALDI-TOF mass spectrometer (Autobio Diagnostics, Zhengzhou, China) and PCR for species and *mcr-1* gene confirmation in transconjugants.

### Protein structure prediction

The three-dimensional structures of the PilV variants were predicted using AlphaFold 3 (43). The full-length amino acid sequence of each PilV variant was submitted independently for structure prediction. The resulting structural models were structurally aligned and visualized using PyMOL, version 3.0.3 (Schrödinger, LLC, New York, NY, USA). All PilV structures were displayed in the same orientation to facilitate comparison of the conserved N-terminal region and the shufflon-derived variable C-terminal region.

### RNA extraction and quantitative reverse transcription PCR

Strains carrying the individual PilV variants were cultured overnight in LB broth at 37°C with shaking at 220 rpm. Overnight cultures were diluted 1:100 into fresh LB broth and grown to the exponential phase. Bacterial cells were harvested by centrifugation, and total RNA was extracted using a bacterial total RNA extraction kit (Vazyme, Nanjing, China) according to the manufacturer’s instructions. RNA concentration and purity were determined using a NanoDrop One microvolume UV–Vis spectrophotometer (Thermo Fisher Scientific, Waltham, MA, USA), and RNA integrity was evaluated by agarose gel electrophoresis. Equal amounts of total RNA were treated to remove residual genomic DNA and reverse-transcribed into cDNA using HiScript III RT SuperMix for qPCR (+gDNA wiper; Vazyme, Nanjing, China) according to the manufacturer’s instructions. Quantitative PCR was performed using ChamQ Universal SYBR qPCR Master Mix (Vazyme, Nanjing, China) on a CFX96 Touch Real-Time PCR Detection System (Bio-Rad Laboratories, Hercules, CA, USA). Melting-curve analysis was performed after amplification to verify the specificity of the PCR products. The *rpoB* gene was used as the internal reference gene. Relative *pilV* transcript levels were calculated using the 2^−ΔΔCt^ method, with the strain carrying PilV-A used as the calibrator. Three independent biological replicates were analyzed for each strain, and each reaction was performed in technical triplicate. The primers used for RT-qPCR are listed in Table S4.

### Statistical analysis

Data are presented as mean ± standard deviation (SD). For comparisons between two groups, unpaired *t*-tests were performed. For comparisons among multiple groups, the Kruskal–Wallis test was performed, followed by Dunn’s multiple comparisons test with correction for multiple testing. Statistical analyses were conducted using GraphPad Prism version 10.5.0 for macOS (GraphPad Software, Boston, MA, USA). An adjusted *P* value < 0.05 was considered statistically significant.

## Data Availability

The publicly available genomic and plasmid sequences analyzed in this study were obtained from the NCBI GenBank database. The genome assembly of *Escherichia coli* ZJ807 is available under accession number GCA_022546355.1 (WGS accession number MCCT00000000; BioSample accession number SAMN05437814). The remaining data supporting the findings of this study are available from the corresponding author upon reasonable request.

## SUPPLEMENTAL MATERIAL

Supplemental tables and figures spectrum.01825-26-s0001.docx Tables S1 to S4 and Figures S1 to S3.

ASM does not own the copyrights to Supplemental Material that may be linked to, or accessed through, an article. The authors have granted ASM a non-exclusive, world-wide license to publish the Supplemental Material files. Please contact the corresponding author directly for reuse.
