# Supplementary material for: Discovery and functional characterization of a novel PilV variant in a mcr-1-positive IncI2 plasmid
Source: Microbiol Spectr. 2026 Sep 30;14(11):e01825-26. doi: 10.1128/spectrum.01825-26 (PMC13729200; doi:10.1128/spectrum.01825-26)
Supplement: Supplementary file 1 [file spectrum.01825-26-s0001.docx]

**Supplementary Material**

**Supplementary Table 1. Number of *pilV* Fragments in Different Host Bacteria.**

| **Host strain name** | **Total strains tested (n)** | ***pilV* fragments** | | | | | | | | |
| --- | --- | --- | --- | --- | --- | --- | --- | --- | --- | --- |
|  |  | A | A' | B | B' | C | C' | D | D' | E |
| *Escherichia_coli* | 388 | 327 | 328 | 57 | 334 | 262 | 261 | 57 | 335 | 113 |
| *Salmonella_enterica* | 46 | 35 | 35 | 10 | 37 | 30 | 28 | 10 | 37 | 12 |
| *Escherichia_fergusonii* | 8 | 8 | 7 | 1 | 7 | 6 | 6 | 1 | 7 | 1 |
| *Salmonella_sp.* | 1 | 1 | 1 | 0 | 1 | 1 | 1 | 0 | 1 | 1 |
| *Klebsiella_pneumoniae* | 17 | 15 | 13 | 0 | 16 | 5 | 5 | 0 | 17 | 0 |
| *Klebsiella_aerogenes* | 2 | 2 | 2 | 0 | 2 | 2 | 2 | 0 | 2 | 0 |
| *Shigella_flexneri* | 3 | 2 | 2 | 0 | 3 | 2 | 2 | 0 | 3 | 0 |
| *Cronobacter_sakazakii* | 1 | 1 | 1 | 0 | 1 | 1 | 1 | 0 | 1 | 0 |
| *Citrobacter_braakii* | 2 | 2 | 2 | 0 | 1 | 2 | 2 | 0 | 1 | 0 |
| *Citrobacter_amalonaticus* | 1 | 1 | 1 | 0 | 1 | 1 | 1 | 0 | 1 | 0 |
| *Shigella_sonnei* | 27 | 27 | 27 | 0 | 27 | 8 | 8 | 0 | 27 | 0 |
| *Shigella_sp._PIB* | 1 | 1 | 1 | 0 | 1 | 1 | 1 | 0 | 1 | 0 |
| *Escherichia_albertii* | 3 | 3 | 2 | 1 | 1 | 0 | 0 | 0 | 0 | 0 |
| *Kluyvera_ascorbata* | 1 | 1 | 1 | 0 | 0 | 0 | 0 | 0 | 0 | 0 |

**Supplementary Table 2. Number of *pilV* Fragments Across Different Isolation Sources.**

| **Isolation Sources** | ***pilV* fragment** | | | | | | | | |
| --- | --- | --- | --- | --- | --- | --- | --- | --- | --- |
|  | A | A' | B | B' | C | C' | D | D' | E |
| unknown | 169 | 163 | 26 | 166 | 128 | 127 | 26 | 166 | 58 |
| *Sus scrofa* | 36 | 35 | 6 | 36 | 30 | 29 | 6 | 36 | 8 |
| *Homo sapiens* | 137 | 140 | 25 | 146 | 101 | 100 | 25 | 145 | 38 |
| *Gallus gallus* | 42 | 42 | 6 | 40 | 31 | 31 | 5 | 42 | 19 |
| *Bos taurus* | 10 | 11 | 1 | 12 | 4 | 4 | 1 | 12 | 0 |
| *Meleagris gallopavo* | 1 | 1 | 1 | 1 | 1 | 1 | 1 | 1 | 0 |
| *Coturnix coturnix* | 1 | 1 | 1 | 1 | 1 | 1 | 1 | 1 | 0 |
| *Prunus dulcis* | 3 | 3 | 2 | 3 | 2 | 2 | 2 | 3 | 0 |
| *Canis lupus* | 7 | 7 | 1 | 8 | 3 | 3 | 1 | 8 | 2 |
| *Aves* | 2 | 2 | 0 | 2 | 1 | 1 | 0 | 2 | 0 |
| *Corvus* | 3 | 3 | 0 | 3 | 3 | 3 | 0 | 3 | 0 |
| *Chordata* | 1 | 1 | 0 | 1 | 1 | 1 | 0 | 1 | 1 |
| *Mus musculus* | 10 | 11 | 0 | 11 | 11 | 11 | 0 | 11 | 0 |
| *Anas platyrhynchos* | 1 | 1 | 0 | 1 | 1 | 1 | 0 | 1 | 1 |
| *Columbidae* | 1 | 1 | 0 | 1 | 0 | 0 | 0 | 1 | 0 |
| *Milvus migrans* | 0 | 1 | 0 | 0 | 1 | 1 | 0 | 0 | 0 |
| *Vulpes vulpes* | 1 | 0 | 0 | 0 | 1 | 1 | 0 | 0 | 0 |
| *Ovis aries* | 0 | 0 | 0 | 0 | 1 | 1 | 0 | 0 | 0 |
| *Columba livia* | 1 | 0 | 0 | 0 | 0 | 0 | 0 | 0 | 0 |

**Supplementary Table 3. Strains and plasmids used in this study.**

| Strain or plasmid | Description* |
| --- | --- |
| DH5α | F–, φ80lacZΔM15, Δ(*lacZYA-argF*), U169, *recA1*, *endA1 hsdR17*(rk–, mk+), *phoA*, *supE44*, λ–, thi–1, *gyrA96*, *relA1* |
| ZJ807 | *E. coli* carrying IncI2 plasmid |
| A42 | *E. coli* carrying IncI2 plasmid |
| A85 | *E. coli* carrying IncI2 plasmid |
| A101 | *E. coli* carrying IncI2 plasmid |
| A434 | *E. coli* carrying IncI2 plasmid |
| A188 | *E. coli* carrying IncI2 plasmid |
| A182 | *E. coli* carrying IncX4 plasmid |
| A211 | *E. coli* carrying IncX4 plasmid |
| A318 | *E. coli* carrying IncX4 plasmid |
| 19ZY283 | *E. coli* carrying IncX4 plasmid |
| 19ZY291 | *E. coli* carrying IncX4 plasmid |
| 19ZY241 | *E. coli* carrying IncHI2 plasmid |
| 19ZY248 | *E. coli* carrying IncHI2 plasmid |
| 21_SD55 | *E. coli* carrying IncHI2 plasmid |
| 21_SD164 | *E. coli* carrying IncHI2 plasmid |
| 21_A206 | *E. coli* carrying IncHI2 plasmid |
| J53 | F^−^, *met*, *pro*, Azi^r^ |
| BW25113 | ∆*(araD-araB)567*, ∆*lacZ4787 (::rrnB-3)*, λ-, *rph-1*, ∆*(rhaD-rhaB)568*, *hsdR514* |
| BW25113/IncI2 | BW25113 strain carrying IncI2 plasmid from ZJ807 |
| BW25113/IncI2 ∆*pilv*-shufflon-*rci* | BW25113 strain carrying IncI2 plasmid::∆*pilv*-shufflon-*rci* |
| BW25113/IncI2 ∆*rci* | BW25113 strain carrying IncI2 plasmid::∆*rci* |
| BL21(DE3) | F–, *ompT*, *hsdSB*(rB-mB-), *gal*, *dcm*, (DE3) |
| ATCC 25922 | FDA strain Seattle 1946; serotype O6, biotype 1; quality-control strain for antimicrobial susceptibility testing |
| *Salmonella* serovar Derby |  |
| DY13 | Ampicillin-resistant recipient strain |
| *Klebsiella pneumoniae* |  |
| 14-124 | Meropenem-resistant recipient strain |
| *Acinetobacter baumannii* |  |
| R64 | Meropenem-resistant recipient strain |
| *Pseudomonas aeruginosa* |  |
| PAO1 | Prototrophic, chl-2, FP−; positive-control strain for biofilm formation |
| 14-113 | Meropenem-resistant recipient strain |
| pET28-His-SUMO-*pilV*-E | pET28-His-SUMO carrying *pilV*-E |
| pET28-His-SUMO-*pilV*-D' | pET28-His-SUMO carrying *pilV*-D' |
| pET28-His-SUMO | *oriV* pBR322; PT7; expression vector (KanR) |
| pACYC184 | Cm^R^ |
| pACYC184-*pilV*-A | pACYC184 carrying *pilV*-A |
| pACYC184-*pilV*-A' | pACYC184 carrying *pilV*-A' |
| pACYC184-*pilV*-B | pACYC184 carrying *pilV*-B |
| pACYC184-*pilV*-B' | pACYC184 carrying *pilV*-B' |
| pACYC184-*pilV*-C | pACYC184 carrying *pilV*-C |
| pACYC184-*pilV*-C' | pACYC184 carrying *pilV*-C' |
| pACYC184-*pilV*-D | pACYC184 carrying *pilV*-D |
| pACYC184-*pilV*-D' | pACYC184 carrying *pilV*-D' |
| pACYC184-*pilV*-E | pACYC184 carrying *pilV*-E |
| pACYC184-*rci* | pACYC184 carrying *rci* |
| pKD3 | Cm^R^ |
| pKD46-APR | pKD46 carrying *aac*(3)-IVa gene |
| pCP20-Spe | pCP20 carrying *aadA* gene |

*Ap^R^, ampicillin resistance; Cm^R^, chloramphenicol resistance; Azi^r^, sodium azide resistance

**Supplementary Table 4. Primers used in this study.**

| Primers | Sequence* | Description |
| --- | --- | --- |
| *pilV*-del-F | TGTTTGTTTACTCACTCGTACCGGGAATATTATTCTGAGGATTAAGAGCAgtgtaggctggagctgcttcg | *pilV*-shufflon-*rci* deletion |
| *pilV*-del-R | TATCCAGAATAAAAAATCACTCTGTAGTTGTTCTATCATAAGTGAATCCTcatatgaatatcctccttag |  |
| ∆*pilV*-yan-F | GCCCATTTCTTTGTGGCGG | *pilV*-shufflon-*rci* deletion verification |
| ∆*pilV*-yan-R | CCTCCGCTGGGAACATTTTG |  |
| RCI-del-F | ATTTCGAAGCAAGAATCTCTACCTTCCCCCCTTTTTTGTCTGGAGGGGATgtgtaggctggagctgcttcg | *rci* deletion |
| RCI-del-R | TATCCAGAATAAAAAATCACTCTGTAGTTGTTCTATCATAAGTGAATCCTcatatgaatatcctccttag |  |
| ∆RCI-yan-F | TTGATCTGCATTGACCGCCT | *rci* deletion verification |
| ∆RCI-yan-R | CTGATAAGCAAACCTACTATCAGGA |  |
| pAC-RCI-F | aacgcagtcaggcaccgtgtATGTTTCAGAAAATCAAAATTCGC | Construction of gene expression vectors used for gene complementation, based on pACYC184 |
| pAC-RCI-R | gaggtgccgccggcttccatCTAATTTGTTAATGGACAAATCATTACC |  |
| pAC-*pilV*-A-F | aacgcagtcaggcaccgtgtATGAAAAAGACAGATAAAGGTGTATCTCT |  |
| pAC-*pilV*-A-R | gaggtgccgccggcttccatTTAAGTTTGGTATCCAAATACTGAAAATAC |  |
| pAC-*pilV*-A'-F | aacgcagtcaggcaccgtgtATGAAAAAGACAGATAAAGGTGTATCTCT |  |
| pAC-*pilV*-A'-R | gaggtgccgccggcttccatTTAATTGAGAGTTACACAGGACGCA |  |
| pAC-*pilV*-B-F | aacgcagtcaggcaccgtgtATGAAAAAGACAGATAAAGGTGTATCTCT |  |
| pAC-*pilV*-B-R | gaggtgccgccggcttccatTTAGCGGAAGCAGTGAACAGTG |  |
| pAC-*pilV*-B'-F | aacgcagtcaggcaccgtgtATGAAAAAGACAGATAAAGGTGTATCTCT |  |
| pAC-*pilV*-B'-R | gaggtgccgccggcttccatTCACTGGCAAATGGCGTAAA |  |
| pAC-*pilV*-C-F | aacgcagtcaggcaccgtgtATGAAAAAGACAGATAAAGGTGTATCTCT |  |
| pAC-*pilV*-C-R | gaggtgccgccggcttccatTTAAATCCCAGCACCAGGAAGA |  |
| pAC-*pilV*-C'-F | aacgcagtcaggcaccgtgtATGAAAAAGACAGATAAAGGTGTATCTCT |  |
| pAC-*pilV*-C'-R | gaggtgccgccggcttccatTTAACCGAAGGGGCAACAATA |  |
| pAC-*pilV*-D-F | aacgcagtcaggcaccgtgtATGAAAAAGACAGATAAAGGTGTATCTCT |  |
| pAC-*pilV*-D-R | gaggtgccgccggcttccatTCACTGACACAATGCATAAGTAGTGAC |  |
| pAC-*pilV*-D'-F | aacgcagtcaggcaccgtgtATGAAAAAGACAGATAAAGGTGTATCTCT |  |
| pAC-*pilV*-D'-R | gaggtgccgccggcttccatTCACTGGCAAACCACGATGTAA |  |
| pAC-*pilV*-E-F | aacgcagtcaggcaccgtgtATGAAAAAGACAGATAAAGGTGTATCTCT |  |
| pAC-*pilV*-E-R | gaggtgccgccggcttccatTTAATCAATACATACTGCATCACAACG |  |
| pAC-V-F | ATGGAAGCCGGCGGCACC |  |
| pAC-V-R | ACACGGTGCCTGACTGCG |  |
| pACyan-F | aattgctaacgcagtcaggc |  |
| pACyan-R | gcattcacagttctccgcaa |  |
| *pilV*-E-F | CGACAGTACAGGGGCGATAC | E fragment verification |
| *pilV*-E-R | CACCGGATTGGCCCCC |  |
| *pilV* protein-D'-F | ccggaggaagtgcaggaattATGAAAAAGACAGATAAAGGTGTATCTCT | Construction of gene expression vectors used for protein expression and purification, based on pET28 |
| *pilV* protein-D'-R | tggtggtggtggtgctcgagTCACTGGCAAACCACGATGTAA |  |
| *pilV* protein-E-F | ccggaggaagtgcaggaattATGAAAAAGACAGATAAAGGTGTATCTCT |  |
| *pilV* protein-E-R | tggtggtggtggtgctcgagTTAATCAATACATACTGCATCACAACG |  |
| pET28-V-F | CTCGAGCACCACCACCACC |  |
| pET28-V-R | AATTCCTGCACTTCCTCCGG |  |
| pET28yan-F | CTCACAGAGAACAGATTGGTGGA |  |
| pET28yan-R | AGCCAACTCAGCTTCCTTTCG |  |
| *mcr-1*-F | AAAGACGCGGTACAAGCAAC | *mcr-1* Verification |
| *mcr-1*-R | GCTGAACATACACGGCACAG |  |
| *PilV*-qp-F | AACAATCACGCCAGCAACAC | qPCR amplification of the *pilV* gene |
| *PilV*-qp-R | CGCCAAGCCCTTCAATCATG |  |
| *rpoB*-qp-F | TCCTTTCTATCCAGCTTGACTCGT | qPCR amplification of the *rpoB* gene |
| *rpoB*-qp-R | CGCAGTTTAACGCGCAGCGG |  |
| E-F | GCTTCCTGCTCCCCTAACG | Sequencing of the E fragment |
| E-R | GGTGGGTAAGCTGTTAGTTGC |  |

*Uppercase letters indicate target-specific annealing regions. Lowercase letters indicate 5′ extensions containing homologous sequences used for Gibson assembly.

**Figure S1. Identification of the PilV-E variant.** (A) PCR of PilV and downstream recombination region fragments. The regulation of the new variant by Rci at the DNA level was verified by PCR. Lane 1: DNA marker; Lane 2: wild-type strain; Lane 3: *rci* knockout strain; Lane 4: *rci*-complemented strain. The amplified bands (primer positions shown as green arrows in Fig. 1) were gel-purified and verified by Sanger sequencing. For primers targeting *pilV*-E, the expected product ranges from 277 bp to 2098 bp. Sequencing primers (E-F/R) are listed in Table S4. (B) Western blot analysis of PilV protein expression. PilV protein levels were analyzed by Western blot. PilV-D' was used as a positive control, and GAPDH served as the internal reference. Protein expression was induced by adding 0.1 mM IPTG to the cultures. The images represent results from three independent experiments.

**Figure S2. Relative transcript levels of *pilV* in strains carrying different PilV variants.** The relative transcription levels of pilV in strains carrying the indicated PilV variants were determined by quantitative reverse transcription PCR (RT-qPCR). Relative transcript levels were calculated using the 2^−ΔΔCt^ method, normalized to the *rpoB* reference gene, and expressed relative to the PilV-A group, which was set to 1. Bars represent the mean ± SD, and individual dots represent independent biological replicates. Statistical analysis was performed using unpaired t-tests, with each PilV variant compared with the PilV-A group. No significant differences were detected (*P* > 0.05).

**Fig. S3. Enlarged views of the C-terminal variable regions of predicted PilV variants.** (A) Structural superposition of all PilV variants; (B) PilV-A; (C) PilV-A'; (D) PilV-B; (E) PilV-B'; (F) PilV-C; (G) PilV-C'; (H) PilV-D; (I) PilV-D'; (J) PilV-E. All C-terminal variable regions are displayed in the same orientation to facilitate comparison among the PilV variants.
